# A review of spatial profiling technologies for characterizing the tumor microenvironment in immuno-oncology

**DOI:** 10.3389/fimmu.2022.996721

**Published:** 2022-10-31

**Authors:** Bian Hu, Muhammad Sajid, Rong Lv, Lianxin Liu, Cheng Sun

**Affiliations:** ^1^Department of Hepatobiliary Surgery, Anhui Provincial Clinical Research Center for Hepatobiliary Diseases, Anhui Province Key Laboratory of Hepatopancreatobiliary Surgery, The First Affiliated Hospital of University of Science and Technology of China (USTC), Division of Life Sciences and Medicine, University of Science and Technology of China, Hefei, China; ^2^Transplant and Immunology Laboratory, The First Affiliated Hospital of University of Science and Technology of China (USTC), Division of Life Sciences and Medicine, University of Science and Technology of China, Hefei, China; ^3^Blood Transfusion Laboratory, Anhui Blood Center, Hefei, China; ^4^Chinese Academy of Sciences (CAS) Key Laboratory of Innate Immunity and Chronic Disease, School of Basic Medical Sciences, Division of Life Sciences and Medicine, University of Science and Technology of China, Hefei, China; ^5^Institute of Immunology, University of Science and Technology of China, Hefei, China

**Keywords:** spatial profiling technologies, transcriptome, proteome, tumor heterogeneity, immuno-oncology

## Abstract

Interpreting the mechanisms and principles that govern gene activity and how these genes work according to -their cellular distribution in organisms has profound implications for cancer research. The latest technological advancements, such as imaging-based approaches and next-generation single-cell sequencing technologies, have established a platform for spatial transcriptomics to systematically quantify the expression of all or most genes in the entire tumor microenvironment and explore an array of disease milieus, particularly in tumors. Spatial profiling technologies permit the study of transcriptional activity at the spatial or single-cell level. This multidimensional classification of the transcriptomic and proteomic signatures of tumors, especially the associated immune and stromal cells, facilitates evaluation of tumor heterogeneity, details of the evolutionary trajectory of each tumor, and multifaceted interactions between each tumor cell and its microenvironment. Therefore, spatial profiling technologies may provide abundant and high-resolution information required for the description of clinical-related features in immuno-oncology. From this perspective, the present review will highlight the importance of spatial transcriptomic and spatial proteomics analysis along with the joint use of other sequencing technologies and their implications in cancers and immune-oncology. In the near future, advances in spatial profiling technologies will undoubtedly expand our understanding of tumor biology and highlight possible precision therapeutic targets for cancer patients.

## 1 Introduction

**Figure 1:**
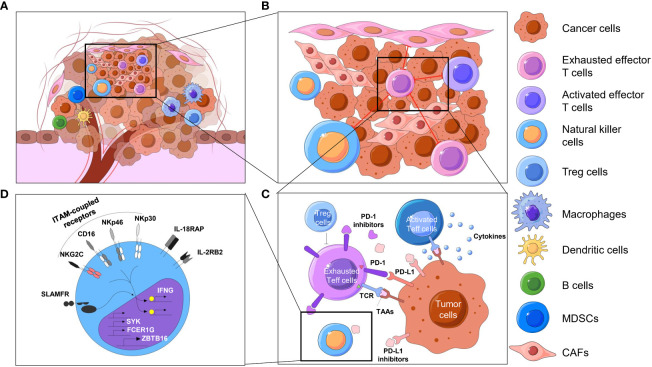
Spatial context of TIME. Overview of the spatial context of the TME and multiomics profiling of the TIME. **(A)** The cellular compositions of the tumor immune microenvironment, including different kinds of immune cells, including T cells, natural killer cells, dendritic cells, macrophages and so on. **(B)** The spatial organization of cells, such as cell neighbors (e.g., proximity of tumor and immune cells) in the TIME, reflects biological processes at the tissue level. **(C)** T cells, for instance, constantly exchange signals with surrounding cells by secreting molecules (e.g., cytokines, chemokines, growth factors) or by direct ligand−receptor binding on the cell surfaces (e.g., immune checkpoints). The activated or exhausted state of these immune cells is an important aspect of the TIME, which may direct different results of immunotherapy. **(D)** When ligands bind to cell receptors, immune cells respond by processing this signal through complex signal transduction networks and transmit information to the nucleus, where the transcription state is changed in an epigenetic manner. MDSCs, myeloid-derived suppressor cells. CAF, cancer-associated fibroblast.

Over the past few centuries, the basic concept of tumors has evolved from a cluster of abnormally proliferating cells to a highly organized “organ” (1, 2). Tumors are intricate combinations of malignant cells, stromal cells, and immune cells. These cells, often with considerable heterogeneity, form the so-called microenvironment (TME), which contains a mixture of antitumor and tumor-promoting signals capable of regulating tumor growth and influencing tumor evolution. Although the particular composition of the TME varies by types of tumors, common features, including various immune cells and stromal cells, as well as the surrounding blood vessels, extracellular matrix (ECM) and soluble factors, have been shown by previous studies (3–6). These immune components that constitute the tumor immune microenvironment (TIME) have been brought to the forefront due to their significant roles in tumor biology, which include innate immune cells such as NK cells and innate lymphoid cells (ILCs), adaptive immune cells such as T cells and B cells, and signaling molecules or factors such as checkpoint receptors and their corresponding ligands on the cell surface or in extracellular regions (7, 8). To discover distinctive information about the TIME, early research extends from the molecular characteristics and compositions to the spatial association and architecture of these factors. The spatial profiling of the TIME is mainly summarized as the following four characteristics: spatial distribution and proportion of diversified immune cells in the tumor compartment (**Figure 1A**); distances between immune cells and their nearest functional-related neighbors (**Figure 1B**) (9); spatial patterns of direct cell-cell interactions at the level of antigen recognition, as well as autocrine and paracrine signaling (**Figure 1C**) (10, 11); and the activated or suppressed state of immune cells with molecular and morphological characterization (**Figure 1D**) (12). While some earlier studies have provided original views on the component and spatial structure of TIME by traditional approaches, advanced techniques have delineated TIME at a considerably elevated degree of throughput, dimensionality and resolution. Evolutionary knowledge and views of the TIME encourage improvements in clinical prognosis and immunotherapy benefits (13–15).

In this review, we retrospectively review some of the current established spatial profiling technologies of the tumor immune microenvironment in the context of tumor immune oncology. Recent advanced profiling technologies, such as transcriptomic and proteomic analyses, along with joint sequencing technologies, will be outlined (**Table 1**). Spatial technology applications in different cancer diagnoses and in immune heterogeneity descriptions will also be comprehensively covered. Finally, a discussion, as well as suggestions for ways to overcome current difficulties and for further improvement, will be given.

**Table 1:** Comparison of spatial sequencing technologies.

Category	Name	Techniques	Description	Reference
**Spatial Transcriptomics Analysis**	Solid phase-based capture	ST/10x Visium	RNA capturing on slides through oligo-dT overhangs and followed by RNA-seq Tissue sections placed onto an emulsion of DropSeq beads for RNA capture, barcoding with 10um resolution	(16)
		Slide-seq	Tissue sections placed onto an emulsion of DropSeq beads for RNA capture, barcoding with 10um resolution	(25, 26)
		High Definition Spatial Transcriptomics (HDST)	uses an Illumina bead array for RNA capture with 2um resolution	(27)
		GeoMx Digital Spatial Profiling (DSP)	UV-cleaved oligo-conjugated RNA probes and barcodes counting or NGS readout with resolution as fine as 10um	(28)
	Selective barcoding method	RNAscope	Hybridization of branched DNA probes followed by signal amplification	(42–45)
	Imaging based in situ transcriptomics	seqFISH	multiplexed smFISH with tens of thousands of distinct transcripts detected	(46–47)
		MERFISH	Applicable to cells and tissue sections; high-resolution	(48)
		*In Situ* Sequencing (ISS)/FISSEQ	Template circularization, RCA and SOLiD-seq	(52, 53)
Spatial Proteomic Analysis	Mass Spectrometric detection	Mass spectrometry	Detection and quantification of protein. lacks adequate sensitivity associated with single-cell analysis	
		Flow cytometry	Evaluate expression of protein at the level of single-cell resolution, even though the number of epitopes that can be detected simultaneously is inadequate by the spectral overlap between fluorophores
		Flow cytometry	Evaluate expression of protein at the level of single-cell resolution, even though the number of epitopes that can be detected simultaneously is inadequate by the spectral overlap between fluorophores
		Mass cytometry	Illustrate particular phenotypes of cells engaged in the response to immunotherapy at single-cell level and capable to distinguish between different reporters	(61)
		Mass spectrometry by Time-of-flight	Combination of high mass resolution, highly compatible with other techniques of selection, easy to design and handle	(66, 67)
		Imaging mass cytometry	used to retrospective analysis of patient cohorts with known outcomes, ultimately helping personalized medicine	(68, 69)
Nucleic Acid Probe based fluorescent Imaging analysis		Point Accumulation In Nanoscale Topography (PAINT)	utilized for super-resolution imaging of recombinant lipid membranes with diffusible dye molecules	(78–80)
		Probe-based Imaging for Sequential Multiplexing (PRISM)	employed to quantify phenotypes and increase imaging throughput.	(77, 78)
The Joint Use of Other Sequencing Technologies	Multi-model Analysis		accomplished through combining multimodel and temporal patterns to obtain information associated to cell ordering at different stages of differentiation	(87–95)

## 2 Spatial transcriptomic analysis

### 2.1 Solid phase-based capture methods

The concept “spatial transcriptomics (ST)” comes from the cross-section of imaging and sequencing brought forward by the groups of Jonas Frisén from the Karolinska Institute and Joakim Lundeberg from National Genomics Infrastructure in Sweden. They were the first to insert the positional barcodes with oligo(dT) primers in the complementary DNA (cDNA) synthesis reaction on glass slides (16), on which they deposited ~200 million oligonucleotides over a 6.2 mm by 6.6 mm square. Tissue sections of the mouse olfactory bulb were immobilized on this area and stained with hematoxylin and eosin for imaging. After that, the tissues were permeabilized and removed. The released RNA was captured, reverse-transcribed *in situ* and subsequently sent for RNA-seq analysis, and aligned with the tissue image according to the positional barcode array features. The cDNA library generated on slides and the preserved spatial information jointly demonstrated spatial transcriptomic heterogeneity while retaining histological context (48). By applying the method to breast cancer biopsies, they found unexpected heterogeneity which was undetected by traditional transcriptomics approaches (49). In addition to their work, data analysis of prostate cancer (49) and melanoma (50) biopsies by ST have also revealed the differences between the tumor area and peripheral region, as well as the intra- and intertumoral heterogeneity. Nevertheless, the resolution of the ST technique cannot reach the single-cell level and is only suitable for fresh frozen samples because of comparatively low readouts (51).

Based on the primary concept of ST, Visium Spatial Gene Expression Solution, which was developed and popularized by 10× Genomics, has become a kit-based assay requiring much less complicated processes. According to the manuscript provided by 10× Genomics, sectioned samples were placed on commercially processed slides in two or four capture squares. These capture squares contain over 5000 spots with millions of oligonucleotides. Each spot is 55 μm in diameter and 100 μm apart from the center to another. The mRNA released from fresh frozen tissues is captured by those oligonucleotides in the spots and a reverse transcription reaction is performed, thus millions of copies of cDNA are generated for next-generation sequencing (NGS). Considering the size and the arrangement of spots, Visium can reach a resolution of 1 to 10 cells per spot. With a higher resolution and enhanced sensitivity, it is now a well-established commercial platform and service for phenotype investigation. Benefiting from its ability to identify aberrant or novel gene expression in the regions of interest, more genes will undoubtedly be found and lead the direction for immunotherapy (52). The Visium software programs for data analysis provided by 10× Genomics make the whole process readily integrated into existing lab infrastructure and require standard equipment, including cryosection stations, standard fluorescence microscopes, and PCR machines. Additionally, by releasing mRNA in formalin and utilizing whole transcriptome probe panels to hybridize to their target genes, formalin-fixed paraffin-embedded (FFPE) samples are also suitable for library construction (52).

The Visium ST technique has been applied to large areas of research and tissues. By applying Visium to human neurological disorder studies, Maynard et al. defined the spatial profiling gene expression in the dorsolateral prefrontal cortex and identified several layer-enriched genes in distinct cortical layers (53). Recently, Maxime et al. used Visium to investigate the response of B cells in tertiary lymphoid structures (TLSs) and found that renal cell carcinoma patients with more intratumoral TLSs had a high response rate to immune checkpoint blockade and prolonged progression-free survival (PFS) (54).

However, the following limitations should be noted. First, the matrix designed on a 6.5 × 6.5 mm square for mRNA capture limits the comprehensive study of the TIME on a large scale and makes the region of interest (ROI) selection subjective based on the users’ experience. Second, because tissue cryosection and permeabilization conditions vary between tissues, species and laboratories, experienced technicians and proper optimization are still needed. Users are recommended to run an optimization experiment for every new tissue type. Third, although FFPE tissues are currently compatible, the quality of the sections still needs to be verified according to our experience. Because of crosslinking and RNA fragmentation, FFPE treatment reduces the detection efficiency as well as the number of genes to approximately 5 to 10 times lower than their frozen counterparts (55). Finally, despite improved resolution compared to ST, 10× Visium still cannot reach single-cell spatial resolution in most samples. Two recently developed techniques named Slide-seq and high-definition spatial transcriptomics (HDST) have improved the spatial resolution of solid phase-based capture to 10 μm and even 2 μm, respectively, by employing beads harboring barcoded DNA probes (17–19). However, the limitation still exists. Slide-seq is currently incompatible with histological imaging, while HDST has limited numbers of unique molecular identifiers (UMIs) and consequently moderate throughput capacity.

### 2.2 Selective barcoding methods

Another commercial ST technique, digital spatial profiling (DSP), is a highly complex approach for obtaining spatiotemporal information. Released by NanoString, GeoMX DSP uses oligonucleotide detection to quantitatively analyze gene expression in frozen or FFPE samples. Instead of crosslinking barcoded oligonucleotides and slides as solid phase-based capture strategies, DNA oligos with barcodes are conjugated to primary antibodies or RNA probes by a special linker. This linker is responsive and cleavable to UV light (20). After proteinase K digestion, the tissue sections are incubated with a customized RNA probe cocktail for RNA profiling. After that, fluorophore-tagged antibodies recognizing different cellular compartments or cell markers (up to four markers) are used to obtain morphological images of tissues. Users can thus select the ROI for further analysis with any determined contours. Once the ROI is selected, the oligos in this region are cleaved by UV light and released and further automatically gathered in tubes, which are transferred to a microtiter plate for further readout by nCounter or NGS. Then the sequencing data will be matched with previous morphological images, providing a comprehensive understanding of the spatial information.

Since this technique does not require scanning a whole slide but just collecting the samples from the ROI, DSP offers a more efficient strategy to generate results from more than 10 tissue sections and up to 384 ROIs in 2 days (20). Researchers have used DSP to quantify PD-L1 expression in a standardized cell line index tissue microarray (TMA) and found high concordance with other routinely used techniques, with high reproducibility and appropriateness for long-term stored slides (56). In addition, as GeoMX uses predefined panels instead of oligo(T) capture, NanoString provides different gene panels with over 1,800 genes or 18,000 genes (20, 57) and can detect up to 96 proteins compared to RNA-profiling-only technologies such as Visium. This advantage expands the contents that we can obtain from one valuable tissue and is notably relevant for evaluating posttranscriptional regulation and posttranslational modifications when mRNA and protein expression patterns are not matched (52, 56). DSP is also particularly attractive for researchers, as it preserves the integrity of tissue samples for further application.

Some limitations of DSP are also evident. First, the selection of the ROI is subjective and may lead to biased hypothesis-driven sample analysis (58, 59). Second, the spatial resolution of GeoMX cannot reach the single-cell level but is several hundred micrometers across (60). Third, the kit provided by DSP can only support 4 colors simultaneously, which restricts the throughput of studies on more details of morphology, such as the spatial organization of different kinds of immune cells in the TME concurrently (58). However, it can be foreseen that with the development of imaging approaches, more channels of fluorescence can be expanded for ongoing use.

### 2.3 Image-Based *in Situ* Transcriptomics methods

*In situ* hybridization (ISH) is a classic technique to visualize target DNA or RNA molecules in cells or tissues, including *Xenopus laevis* oocytes and *Drosophila*, for developmental studies (61–64). Since radioactive-, fluorescent- or colorimetric-labelled nucleic acid probes and their hybridized onto targets were all developed in the context of ISH (64–66), it can be seen as a precursor of current “spatial transcriptomics” techniques. With fluorescently labelled probes widely used for detecting microorganisms and diagnosing solid or hematological cancers, fluorescence *in situ* hybridization (FISH) has become a routine clinical test (67, 68). The relatively low throughput, however, has limited conventional FISH to describe the heterogeneity of the TME and TIME.

Since 2013, several improved techniques have been developed based on ISH, such as RNAscope, high-throughput ISS (*in situ* sequencing), seqFISH and multiplexed error-robust FISH (MERFISH) and those methods based on them. Representative of single-molecule FISH (smFISH), RNAscope (Advanced Cell Diagnostics, USA) is a commercialized ISH-based technology that can distinguish up to 12 different RNA targets each time and achieve better sensitivity and specificity than conventional FISH, even when the target is low in abundance (21–24). Subsequently, multiplexed smFISH was developed to increase the resolution of mRNA detection on ROIs, such as sequential FISH (seqFISH), which could enlarge the volume of target genes from 12 in fixed cells to over 10,000 in sections (25, 26), or MERFISH (27), which applied 4 sets of overhanging probes for fluorescence labelling and acquired images with decoded color-dependent readout patterns. Multiple rounds of hybridization were performed to increase the brightness of the fluorescence signal as well as the numbers of mRNAs to be detected (69, 70). This system was recently released by Vizgen as a commercial MERSCOPE platform.

ISS is another image-based method using barcoded padlock probes to align with the cDNA reverse transcribed *in situ*, followed by circularization and target-primed rolling circle amplification (RCA) (71). Several detection and sequencing methods relying on ISS have been successfully established, such as FISSEQ (fluorescence *in situ* sequencing) (28, 29) and STARmap (spatially-resolved transcript amplicon readout mapping) (72). Nonetheless, these methods are limited by the length of cDNA synthesized *in situ*, and the difficulty of spatially mapping regulatory elements in a systematic as well as unbiased manner. Two recent methods, *in situ* transcriptome accessibility sequencing (INSTA-Seq) and Expansion sequencing (ExSeq), were reported to resolve the problem by combining ISS with ex situ sequencing (73, 74), indicating a new strategy for depth expansion in spatial information acquisition.

## 3 Spatial proteomic analysis

### 3.1 Mass spectrometric-based detection

At present, spatial transcriptomics and spatial proteomics are the two most frequently applied spatial omics techniques for *de novo* studies or revealing the landscape of the TIME. Proteins signify the primary operational mechanisms of cells, and thus decoding of the proteome expressed at the single-cell stage is of great importance (2). Mass spectrometry is essential for the detection and quantification of proteins. Nevertheless, it is only available for abundant proteins. Investigators have strengthened methods for the formulation and sequestration of proteins, diminishing the loss of proteins and facilitating deeper quantitative proteomic sequencing at single-cell resolution (75). Single-cell proteomic analyses mainly rely on the recognition of targets by employing antibodies or analogous affinity elements, including aptamers or affibodies (76–78). The initiation of mass cytometry requires the application of labelling antibodies with metal isotopes, which considerably increases the quantity of markers that can be examined concurrently on single cells (30). For instance, Wagner et al. generated a comprehensive single-cell atlas of breast cancer using a 35-marker immune cell-centric panel and a 38-marker tumor cell-centric panel (79).

Mass cytometry is a specifically effective method to illustrate particular cell phenotypes engaged in the response to immunotherapy at the single-cell level, as shown in the findings of two studies of melanoma patients’ response to immune checkpoint inhibitors (ICIs) (80). In one study, the researchers utilized mass cytometry to explain that an elevated occurrence of classical monocytes circulating prior to cure is a robust forecaster of anti-PD-1 antibody response, as demonstrated by the analysis of data from a validation cohort of thirty-one patients employing flow cytometry (81). In another investigation, Gide et al. analyzed melanoma biopsies acquired from a subgroup of patients who received anti-PD-1 antibodies as monotherapy or in a mixture with anti-CTLA-4 antibodies. Dimensional decline assessment of a 43-marker large-scale flow cytometry panel recognized fourteen immune cell groups consisting of 3 distinctive T-cell groups. The important markers of differentiation, activation and exhaustion were observed co-expressed on CD45RO+EOMES+ T-cell population, together with the markers of activation and tissue resident (CD69), tissue resident memory (CD103) and effector memory (HLA-DR, T-BET and low CCR7). This subpopulation expresses the inhibitory receptors PD-1 and TIGIT, but low levels of CD57, suggesting that they are not terminally exhausted (82).

Mass cytometry by time-of-flight (CyTOF) depends on the immunolabelling of isotope-labelled antibodies that bind to certain signaling molecules on the cell surface or within the cell, allowing the analysis of 100 different proteins in a single cell (31, 32). Imaging mass cytometry (IMC) was established based on immunohistochemistry with metal-labelled antibodies and CyTOF. IMC can concurrently investigate up to 40 markers of proteins, as well as their spatial structure and connections, which is impossible when using conventional lysis of tissue into single cells. Crucially, IMC can also be applied to paraffin-embedded tissue sections, so it can be used for retrospective analysis of patient groups with known results, ultimately helping with personalized medicine (33, 34). Recently, SCoPE-MS (single-cell proteomics mass spectrometry) based on tandem mass spectrometry (MS/MS) has been established to compute multiplexed single-cell proteomes (83, 84). Single-cell proteomic sequencing technology will change the context of exploring single cell, especially once incorporated into multi-omics technologies.

### 3.2 Nucleic acid probe-based fluorescence imaging analysis

Multiplexed protein imaging approaches for overcoming the spectral limitations of traditional fluorescence microscopy normally require several cycles of antibody staining and imaging, attained by either elution of antibodies or inactivation of fluorophores utilizing photo- or chemical bleaching (85, 86). In contrast, using diffusive fluorescent imaging probes targeting particular markers of protein or *in situ* antibodies incapacitates the above boundaries by providing (1) fast probe exchange by employing minor buffering, (2) instantaneous antibody loading prior to imaging, and (3) super-resolution imaging by employing point accumulation in nanoscale topography (PAINT) (87). PAINT was initially proposed by Hochstrasser and Sharonov (88) and was initially utilized for super-resolution imaging of recombinant membranes of lipids with diffusible dye elements. Later, numerous applications and variants of this method were established to produce multichannel data (38, 89), which includes uPAINT (35) using DNA-PAINT and diffusible fluorescent antibodies (86, 87) that make use of df-ssDNA (diffusible fluorescent single-stranded DNA) molecules (imaging probes) that rapidly combine with corresponding ssDNA oligos (docking strands) affixed to target DNA antibodies (89) or nanostructures (38).

Protein-based fragment probes can also be employed to generate multiplexed cytoskeleton and central adhesion super-resolution images with higher labelling intensities than antibody-based methods (85). However, this approach involves the detection of extremely specialized, transiently bound peptides for every single target molecule, which can be difficult to simplify to additional proteins, especially those expressed at reduced amounts compared to proteins of the cytoskeleton. Remarkably, each of the above super-resolution methods depends on time utilization and possesses short-throughput time delay imaging resulting from fluorophore reconstruction and localization used in the traditional single-molecule localization microscopy methods STORM (36) and PALM (37). In addition, diffusible fluorescent probes often generate high background fluorescence, preventing them from quantitative phenotyping of neuron cells employing high-throughput confocal imaging of drug and genomic agitations (90). Probe-based Imaging for Sequential Multiplexing (PRISM) has been employed to quantify phenotypes and increase imaging throughput. Fluorescently labelled single-stranded locked nucleic acid ssLNA (for LNAPRISM) and traditional ssDNA (for DNA-PRISM) oligonucleotides can be used alternately as low- and high-affinity imaging probes for diffraction-restricted confocal or PAINT-based super-resolution imaging. Longer, higher-affinity ssDNA imaging probes could in principle be used for confocal imaging, as previously described (86, 87). A previous study applied LNA-PRISM to thirteen-channel confocal imaging of 7 synaptic proteins simultaneously imaged in cultures of neuron cells, of which five cytoskeletal proteins have been demonstrated to cooperate with each other *in vivo* (91). These multiplexed imaging data facilitate measurable analysis of sixty-six protein co-expression profiles obtained from thousands of distinct synapses inside the identical intact neuron, showing intense associations between subsets of synaptic proteins, as well as discrepancies in synapse subtypes. Furthermore, a previous study utilized LNA-PRISM to measure fluctuations in the levels of synaptic proteins and the composition of synaptic subtypes in excitative synapses following obstruction of voltage-gated sodium channels with tetrodotoxin (TTX) treatment, as previously described, to observe the mechanisms of homeostatic plasticity (92–95).

Probe-based *in situ* techniques are considered for large-scale purposes through their comprehensive maintenance of spatial evidence and comparatively minimal expense. However, probe-based techniques only use existing probes to detect known targets. Therefore, they are not suitable for identifying new biological effects, such as indeterminate molecules.

## 4 The joint use of other sequencing technologies

There is increasing interest in integrating different single-cell analysis modalities into comprehensive techniques. Single-cell multimodal analysis can also be used to describe how cellular states evolve along different stages of cellular differentiation. This can be achieved by combination of multimodal and temporal patterns to capture information related to cell ordering at different stages of differentiation (92). Four different strategies can be employed to achieve multimodal data. First, using a non-destructive assay such as multiparameter fluorescence-activated cell sorting (FACS) prior to sequencing, which can allow the measurements of protein levels linked to the transcriptomes in the same cell; second, the separation of different cellular fractions or components for parallel experiment in lyse-and-split way. For example, the nucleus can be physically isolated for bisulfite sequencing while the rest cytosolic mRNAs can be used for scRNA-seq, which will gather data on the methylomes and transcriptomes respectively; third, converting multimodal data into a common molecular format, like cDNA, for simultaneous analysis by common methods such as DNA sequencing. For example, cell surface protein can be captured by antibodies conjugated with polyadenylated barcodes. Combined with standard scRNA-seq, the polyadenylated barcodes can be captured for an estimate of protein levels for each cell; fourth, additional information extraction from analysis of scRNA-seq data, such as using unspliced intron detection for RNA velocity estimation (93). Technologies that incorporate single-cell recognition of proteins into scRNA-seq procedures, such as REAP-seq and CITE-seq, employ antibodies conjugated to barcoded oligonucleotides *via* a cDNA sequencing step for scRNA-seq, enabling protein detection and thereby not limited by spectral overlap or the amount of solvable metal ions (94, 95). For instance, juxtaposition expansion detection using barcoded antibodies facilitated the improvement of a commercial 96-plex panel for protein detection in plasma and serum samples (39). The expansion of the throughput of protein detection will surely benefit for our knowledge of the TIME from limited samples. The use of oligonucleotide-labelled antibodies is integrated into the Tapestri workflow for the sequencing of single-cell DNA, presenting an uncomplicated procedure of combining phenotype and genotype (40).

The scATAC-seq (single-cell Assay for Transposase-Accessible Chromatin with high-throughput sequencing) data encompass sequencing reads from the mitochondrial genome. Even though these signals were initially seen as an experimental nuisance, this property can detect mutations in mitochondria DNA, which may be employed to achieve clone tracing (41, 42). Consequently, scATAC sequencing allows instantaneous quantification of cell fate (through the nuclear epigenome) and cell lineage (through the mitochondrial genome). Mutations in mitochondria DNA may be identified in scRNA-seq data, albeit less than in scATAC sequencing data owing to less and lower consistent coverage of the mitochondrial genome (43). The tracing assessment of such lineages might be utilized in the future to better identify which clones of tumor cells develop, and such clones may be associated with resistance to treatment.

## 5 Spatial profiling technologies applications

The rapid development of current spatially resolved transcriptomics technologies has extended our view of subjects, from human, mouse, and nonhuman primate brains (27, 44–47), to various cancers (49, 50, 96–99). The growing interest in analyzing the molecular constitution of the TME or TIME and the demand to study cell-cell interactions call for wide use of these technologies in clinical and biological research. Basically, the applications of spatial profiling technologies lie in three aspects: 1) Discovering the heterogeneity of cells in cancers or other diseases, especially on immune cells; 2) Establishing spatial transcriptome atlases for human, mouse or even plant tissues; and 3) Delineating an embryonic developmental and spatial blueprint on human or model animals.

### 5.1 Applications in cancers

To study cancer as a complicated biological system with extensive heterogeneity intratumorally and across patients, single-cell technologies such as flow cytometry or single-cell RNA sequencing have been widely applied and have expanded the depth of research, such as, showing more details of the TIME. However, to investigate the spatial contexts of the microenvironment, image-based techniques such as FISH, Visium and multiparameter immunofluorescence should be combined.

FISH has been widely used to detect oncogenes in surgical pathology, especially on the alterations of chromosomes, including deletions, gains, translocations, amplifications and polysomy. Many mutations, such as the BCR-ABL1 t (9; 22) translocation, EML4-ALK fusion gene and TMPRSS2-ERG fusion gene, have been identified by FISH in chronic myeloid leukemia, non-small cell lung cancer and prostate cancer, respectively (67, 100, 101). Researchers have also used FISH to predict and judge the clinical response to PD-1 blockade therapy in advanced melanomas and renal cell carcinomas (102, 103). By overcoming the limitation of conventional FISH at the DNA level, RNAscope was developed to detect low-content mRNA, avoiding high background, and applied in evaluating the expression of immune checkpoints in cancers for diagnosis, including lung adenosquamous cell carcinomas, advanced gastric cancer, triple-negative breast cancer and bladder carcinomas (104–107). It can also be used in studying the mechanisms of immune escape, new prognostic cancer biomarkers (108–113) or even in tracing genetically modified cells, including CAR-T cells, in human bodies after infusion to provide long-lasting validation (114). Similarly, multiplexed smFISH was also used to study tumor heterogeneity in breast cancer, combined with microfluidics technology (115).

Visium has also been reported to reveal the heterogeneity of cancers. Willis et al. utilized this technique to identify the induction of IL-6 signaling *in vivo* and suggested its therapeutic value (116). For another interesting example, tumor-specific keratinocyte (TSK) clusters were identified with MMP10 as a marker by the first generation of ST at the tumor leading edges in patients with squamous cell carcinoma. With the help of enhanced Visium, enrichment of transcripts related to endothelial and cancer-associated fibroblasts in the stroma was found, revealing a fibrovascular niche surrounding TSK clusters in the TME (98). DSP has also been widely applied in predicting and evaluating the response to immunotherapy, especially immune checkpoint blockade, such as anti-PD-1/PD-L1 therapies (117), which provides standardized, objective and accurate data on the expression of checkpoints of interest within defined locations. Compared to other routinely used techniques, such as quantitative immunofluorescence, high consistency and reproducibility can be seen by DSP (56).

### 5.2 Applications in immunology

The contents of the TIME have been proven to play a crucial role in the progression of tumorigenesis, including metastasis and recurrence. The attempt to transform the tumor-promoting TIME into a tumor-inhibiting environment has encouraged us to focus on exploring the mechanism of the tumor-immune interplay involved in the TIME. A thorough examination has already been carried out, and the details below the phenotypes are to be discovered by relying on new technologies.

**Figure 2:**
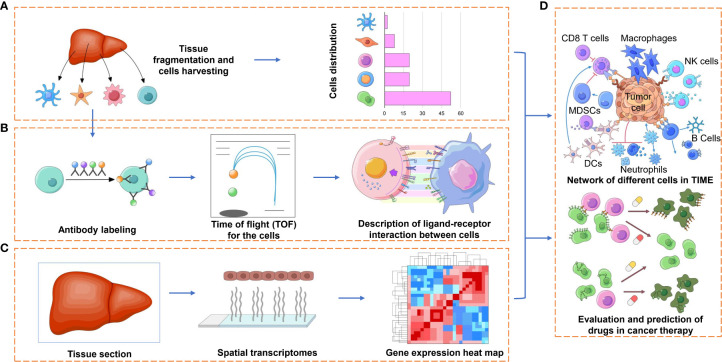
Applications of spatial profiling technologies to the TIME. Representation of some of the application facets of the TIME research by spatial profiling technologies. **(A)** The detection and quantification of the different immune cell types of the TIME, which may have pro- or antitumorigenic roles, can provide prognostic and predictive markers for immunotherapy. **(B)** Characterizing various cell subtypes (e.g., CD4+ and CD8+ T cells, NK cells, macrophages) by different functional orientations (e.g., naïve, effector, memory) and states (e.g., activated, anergic, exhausted) by technologies such as cyTOF, which may lend to a comprehensive understanding of the immense diversity of ligand receptors between tumor cells and immune cells or to finding more inhibitory receptors on immune cells. **(C)** The spatial and transcriptional information provided by ST or Visium with further bioinformatic analysis will be used for the description of TIME in a high-throughput way. **(D)** Evaluation and prediction of the curative effect of cancer therapies, such as immunotherapy with immune checkpoint blockade or targeted therapy, based on the combined information provided above.

On the other hand, it is well known that immune cells and their secreted factors are the main components of the TIME. The former, which involves T cells, B cells, natural killer (NK) cells, macrophages, dendritic cells (DCs), myeloid-derived suppressor cells (MDSCs) and so on (**Figure 2A**), have complex, disabling or even opposite functions compared to their normal state. The latter, including cytokines, chemokines and growth factors (**Figure 2B**), also show benefits in clinical application (refusion or blockade), while the toxicity and which to choose for use still need to be addressed. Analyzing immune elements in a reductionistic way may lead to confusion and misunderstanding. A comprehensive understanding of these elements through the application of high-throughput technologies such as single-cell transcriptomics, proteomics, epigenomics, and bioinformatics can provide novel insights and better strategies for cancer immunotherapy.

#### 5.2.1 Characterization of immune cell heterogeneity in the TIME

Traditional bulk gene profiling cannot distinguish RNA profiling from tumor cells or immune cells. Classic single-cell RNA-seq could solve the problem but could not reveal the localization of these immune cells. Spatial technology approaches are valued for the following reasons: first, cell populations characterized by several typical biomarkers can be qualitatively analyzed across the spatial dimension or in subcellular resolution; second, visualization in the ROI field after image processing and analysis could characterize the state of immune cells across the whole biopsy sample and is of diagnostic benefit to clinical research; third, tracking the distribution of cells of interest, for example CAR-T, in some specific regions or tissues (**Figure 2C**).

Jeyasekharan et al. used a customized panel of 36 antibodies for DSP from samples of diffuse large B-cell lymphoma (DLBCL) patients pretreated with chemoimmunotherapy (118) and revealed that CD68+CD163+ M2 macrophages showed dramatic negative impacts on prognosis. This finding provides the basis for further treatments targeting tumor-infiltrating macrophages. Helmink et al. used a multiplex immunofluorescence assay and scRNA-seq to analyze the responses of intratumorally B cells in metastatic melanoma and renal cell carcinoma (RCC) after immune checkpoint blockade. They have shown the supporting role of B cells in T-cell function within tertiary lymphoid structures (TLSs), and differential B-cell phenotypes, including memory B cells, double-negative (CD27-IgD-) subtypes, and plasma (-like) cells, were found by mass cytometry (119). Cabrita et al. also used metastatic melanoma samples and GeoMx DSP with proteomic analysis to reveal that different mature stages of TLSs existed in tumors, and mature TLSs were associated with high expression of Ki67 and CD40 on B cells, as well as a high proportion of CD4+ T cells and increased memory T cells. This signature could be used for predicting the response to ICBs (120).

In short, spatially resolved high-plex profiling technologies have enhanced our ability to study the interactions and relationships of the contents in the TIME to a much deeper and finer degree than ever and will benefit immunotherapy for various tumors (**Figure 2D** up).

#### 5.2.2 Identifying biomarkers or neoantigens for immunotherapy

It is becoming common practice that expanded autologous or allogeneic immune cells, genetically engineered or not, are infused into patients with tumors for so-called adoptive cell therapy (ACT) (121). The infused cells include engineered TCR-T cells and chimeric antigen receptor T cells (CAR-T cells) or CAR-NK cells in which T or NK cells are armored by artificial TCRs or CARs to recognize tumour-associated antigens (TAAs). Given that T effector cells and NK cells may be lost of function or inhibited in the TIME, engineered TCRs or CARs provide a specific activator for these immune cells. Researchers and pharmaceutical enterprises have long been searching for appropriate and effective TAAs, which are consistently expressed on tumor cells but are not or less expressed on healthy tissues. Therefore, the identification of neoantigens or biomarkers will benefit ACT directly (**Figure 2D** below).

It has been shown that TAAs searched from colorectal cancer, breast cancer, and cholangiocarcinoma could be beneficial for T-cell recognition and killing of tumor cells *in vitro* (122–124). Tran et al. identified 26 somatic mutations from a metastatic cholangiocarcinoma patient and transferred these mutant genes into APCs for further antigen presentation. After coculturing these APCs with patient-derived TILs, antigen-specific CD4^+^Vb22^+^ T-cell clones were identified, isolated and proven to be effective in epithelial cancer (122). There is no doubt that once combined with spatial transcriptomics technologies, more neoantigens will be found or confirmed in biopsy samples.

## 6 Conclusion and future perspectives

Spatial profiling techniques have led to a whole range of discoveries about the mechanisms underlying the immune system. We highlighted some of these findings, which provide awareness of the spatial characteristics of different technologies and their applications in the field of immunology. These technologies are becoming more accessible and applicable in the diagnosis of different cancers and will be employed to meet increasingly complex and multidimensional requirements, such as computing relationships between proteins and their corresponding transcripts or analyzing the landscape of posttranslational modifications. New multimodal single-cell spatial profiling techniques are proposed every year, some of which have not yet been applied to immune cells. For instance, a significant but unanswered problem is the identification of target genes associated with transcription factors, as this leads to a better interpretation of the molecular network modules that operate immune cell pedigrees and their differentiation. Techniques for assessing spatial profiling are advancing rapidly, so there is no single spatial profiling technique that is considered best for all purposes. Based on the biological problem posed, experimental approaches can be developed to combine any spatial profiling methodology with single-cell RNA-seq. In summary, the integration of sequencing technologies can yield more complicated, high-throughput data, including genomic, transcriptomic, epigenomic, proteomic, spatial and temporal information, which requires the expansion of robust and accurate algorithms or models that stipulate novel approaches for classification, diagnosis, prognosis prediction, and targeted therapy. Conspicuously, the sensitivity and precision of emerging technologies and computational analysis must be improved, so that costs will become more reasonable in the near future.

## Author contributions

CS conceived and directed the project. BH and MS contributed equally. BH, MS, and CS drafted the manuscript. All authors contributed to the article and approved the submitted version

## Funding

This work was supported by the National Key Research and Development Program of China (# 2021YFC2300600), National Natural Science Foundation of China (#82022056, #92169118, #91942310) and Natural Science Foundation of Anhui Provincial (# 2008085J35).

## Conflict of interest

The authors declare that the research was conducted in the absence of any commercial or financial relationships that could be construed as a potential conflict of interest.

## Publisher’s note

All claims expressed in this article are solely those of the authors and do not necessarily represent those of their affiliated organizations, or those of the publisher, the editors and the reviewers. Any product that may be evaluated in this article, or claim that may be made by its manufacturer, is not guaranteed or endorsed by the publisher.
